# Monitoring of Large Diameter Sewage Collector Strengthened with Glass-Fiber Reinforced Plastic (GRP) Panels by Means of Distributed Fiber Optic Sensors (DFOS)

**DOI:** 10.3390/s21196607

**Published:** 2021-10-03

**Authors:** Paweł Popielski, Bartosz Bednarz, Rafał Sieńko, Tomasz Howiacki, Łukasz Bednarski, Bartosz Zaborski

**Affiliations:** 1 Faculty of Building Services, Hydro and Environmental Engineering, Warsaw University of Technology, Nowowiejska Street 20, 00-653 Warsaw, Poland; bartosz.bednarz@pw.edu.pl; 2 Reinforced Concrete Structures Division, Faculty of Civil Engineering, Cracow University of Technology, Warszawska Street 24, 31-155 Cracow, Poland; rafal.sienko@pk.edu.pl (R.S.); tomasz.howiacki@shmsystem.pl (T.H.); 3 SHM System Sp. z o.o., Sp. kom., Libertów, ul. Jana Pawła II 82A, 30-444 Krakow, Poland; 4 Mechanics and Vibroacoustics, Faculty of Mechanical Engineering and Robotics, AGH University of Science and Technology in Krakow, Mickiewicza Al. 30, 30-059 Krakow, Poland; lukaszb@agh.edu.pl; 5 MPWIK SA (Municipal Water Supply and Sewerage Company in Warsaw Joint Stock Company—Bureau of Research and Technology), Starynkiewicza 5 Street, 02-015 Warsaw, Poland; b.zaborski@mpwik.com.pl

**Keywords:** distributed fiber optic sensing DFOS, strains, cracks, displacements, collector, tunnels, concrete, glass fiber reinforced polymer GFRP, strengthening, layered cables, monolithic sensors

## Abstract

Diagnostics and assessment of the structural performance of collectors and tunnels require multi-criteria as well as comprehensive analyses for improving the safety based on acquired measurement data. This paper presents the basic goals for a structural health monitoring system designed based on distributed fiber optic sensors (DFOS). The issue of selecting appropriate sensors enabling correct strain transfer is discussed hereafter, indicating both limitations of layered cables and advantages of sensors with monolithic cross-section design in terms of reliable measurements. The sensor’s design determines the operation of the entire monitoring system and the usefulness of the acquired data for the engineering interpretation. The measurements and results obtained due to monolithic DFOS sensors are described hereafter on the example of real engineering structure—the Burakowski concrete collector in Warsaw during its strengthening with glass-fiber reinforced plastic (GRP) panels.

## 1. Introduction

Aging of the existing infrastructure and dynamic development of new infrastructure in the cities are becoming a growing environmental threat to people. The development of large-scale infrastructure, including deep-founded buildings, is causing constant changes in the groundwater regime, which often intensifies their negative impact on susceptible structures. Conversely, urban areas are exposed to a number of additional factors that shorten the operational life of installations, such as stray currents. All these aspects have a negative impact on the condition of water and sewage infrastructure, especially those components of these systems that were built in the previous century, sometimes using low-quality materials. Leaks and failures may result in contamination of the ground around sewage collectors, as well as contamination of underground and inland waters [1]. It is necessary to maintain the infrastructure at a technical level sufficient to guarantee its proper performance and safety. Repair and modernization of the existing underground infrastructure and construction of new sections of the system increase the degree of interaction between these two. Direct access to the structure required to assess its technical condition is becoming increasingly difficult, or in many cases, impossible. With the help of modern measurement technologies, it is possible to obtain a monitoring network with a high measurement resolution and providing information in a continuous and automated manner, which can significantly reduce the need to perform physical inspections of the structure and increase comfort and safety for employees responsible for the performance of water and sewage networks. Important types of facilities where the use of distributed sensors plays an important role are tunnels, sewer collectors, pipelines and adits, where significant stress concentrations can be caused by adjacent infrastructure and varying water and ground conditions.

The article describes the concrete sewage collector originally constructed in 1964 and now reinforced with Glass–fiber Reinforcement Plastic (GRP) panels. Such structural components are made of glass fiber reinforced polyester resins. This technology allows to obtain any shape, fitting it to an existing pipeline or sewer cross section [2].

The space between the panels mounted inside and the existing inner walls of the collector was filled with a cement grout. Fitting self-supporting GRP panels inside the existing concrete structure is a process that completely cuts off any access to the original collector structure, making it difficult to assess the phenomena occurring in the original structure and at the interface between different materials. To better understand the complex state of stresses occurring during the retrofit and further operation, a decision was made to use comprehensive structural health monitoring (SHM), which allows to obtain measurement data and to assess the structural performance of the sewage collector—without the need for additional inspections.

Warsaw’s sewer network is currently nearly 4200 km long, most of which will require renovation in the coming years. The owner of the network is interested in finding modern monitoring solutions which would make it possible to supervise the process of modernizing the network and its future operation. This will allow more effective quality control of the network, reduce the number of potential failures and extend the life of the renovated collectors.

DFOS-based structural health monitoring is pretending to meet the above goals and that is why the pilot installation was conducted within the modernized section of the “Burakowski” sewer collector in Warsaw, Poland.

## 2. Brief Description of the Collector in Question

**Figure 1:**
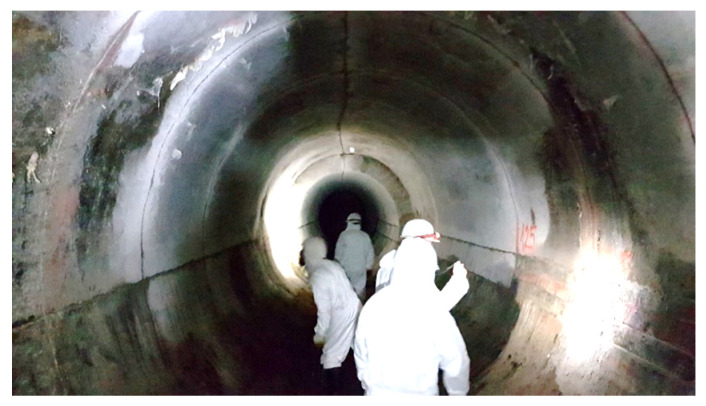
The view of Burakowski concrete collector during its technical inspection.

The Burakowski sewage collector is an important element of the Warsaw sewage system. It was built in the 1960s using the mining method in a concrete casing, with segments of lengths from 2 to 3 m and an internal diameter of approximately 3 m, buried under the ground surface at a depth of 4.5 to 7.5 m (Figure 1). Its purpose is to transport sewage from the part of Warsaw situated on the left bank of the Vistula River to the transmission system that subsequently transports it under the river to the sewage treatment plant. Its calculated capacity is about 12 m^3^/s (the actual recorded capacity of the collector may be higher during heavy rainfall).

There are a number of unfavorable external factors in the collector area. The most important ones include the location near the edge of the high bank of the Vistula River and the vicinity of subway and tram lines and stations. A number of modern, high and deep-founded buildings have also been built in the area over the past 15 years. Moreover, in mid-2015, the construction of a new Burakowski Bis collector was completed in the immediate vicinity, along the existing Burakowski collector, which resulted in a number of changes in the environment of the existing collector compared to when it was originally constructed.

**Figure 2:**
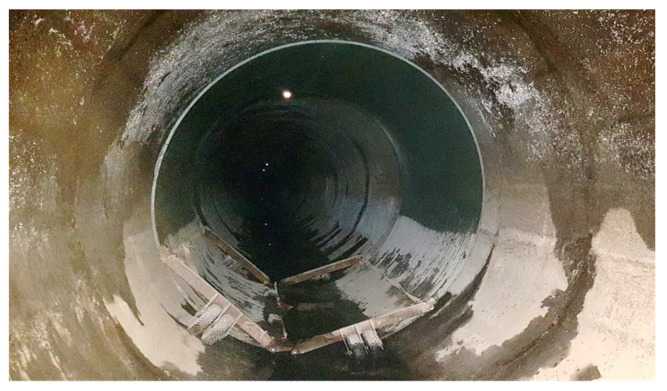
GRP test panel inside a collector before installation.

In 2019, a decision was made to retrofit a 4.8 km long section of the collector using non-circular GRP modules and relining technology [3]. The interior of the collector was lined with 1600 modules of polyester resin panels, each 3 m long (Figure 2). The space between the panels placed inside and the existing inner walls of the collector was filled with a cement grout.

## 3. General Goals for DFOS-Based Monitoring System

Because of the importance of the Burakowski collector in Warsaw’s sewage distribution system and the applied method of its renovation using standard and split self-supporting GRP panels, the following issues requiring verification were identified:

- assessment of the structural performance of the retrofitted collector structure;
- tracking the development of cracks identified during the inspection of the existing concrete casing of the collector;
- behavior of the original collector structure during the renovation works, in particular behavior of the identified cracks and detecting the new ones that occur at key stages of works, i.e., placement of GRP panels inside the collector, gap grouting process, grout setting process;
- monitoring the mating of GRP modules with concrete casing after completed renovation and during the operation of the renovated section, e.g., in extreme operating conditions (when completely filled).

Identification of the abovementioned issues shows the need of application the measurement technique that allows to:

- monitor the collector strains along its entire length (geometrically continuous/distributed measurements—distributed fiber optic sensing (DFOS));
- locate and monitor the development of cracks and fractures (large measuring range);
- fully integrate sensors to the monitored object, i.e., concrete collector casing and grout;
- ensure a possibly high ease of installation, high resistance to the mounting stress as well as harsh environmental conditions.

The objectives established for monitoring the collector’s longitudinal strain dictated the need for geometrically continuous, distributed fiber optic measurements using DFOS sensors. Fiber optic-measurement technology allows to use Rayleigh, Brillouin or Raman scattering-based techniques for measurement. As a result, it is possible to measure both temperature changes (Raman scattering) [4] and strain with geometrical resolution from 5 mm (Rayleigh scattering) as well as strain over long distances (Brillouin scattering) [5].

## 4. Analysis of Available Solutions: Layered Sensing Cables

**Figure 3:**
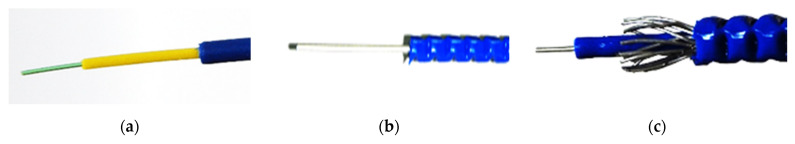
Examples of sensing cable designs: (**a**) with plastic coating; (**b**) with metal coating and single or multiple plastic coatings; (**c**) with metal coating and additional metal reinforcing elements and with single or multiple plastic coatings.

The key task preceding construction of the monitoring system was to select fiber optic elements used to measure strains. A popular and widely used market solution is layered sensing cables. A common feature of all commercially available solutions is their multi layered designs (Figure 3).

The need for an operating principle of coatings in sensing cables is explained in a patent application filed by F. Ravet from Omnisens in 2014 (US 2014/0033825 A1—Method And Assembly For Sensing Permanent Deformation Of a Structure). The patent application provides a description and principle of operation for most of the commercially available sensing cable designs. The primary purpose of the coatings in a sensing cable is to protect the optical fiber from rupture. When the fiber reaches the strain assumed at the cable design stage, particular layers of the sensing cable start to slip against each other. The layers split and shift in relation to each other. This behavior of the sensing cable protects the optical fiber from breakage by reducing its strain and, at the same time, it mechanically records the occurring event. Mechanical shifting of the layers sustains the tension of the fiber when external impacts cease, which makes it possible to detect exceeding of the assumed strain threshold at any time (the sensing cable has mechanical memory of exceeding the strain level determined at the stage of cable design). In order for layered sensing cables to fulfill their role, they must be designed and manufactured individually for each system, considering the location of sensors and the expected strain level such that the slippage between the layers of the sensing cable occurs at a precisely defined strain value. This means that it is not possible to use general purpose layered cables for different applications.

**Figure 4:**
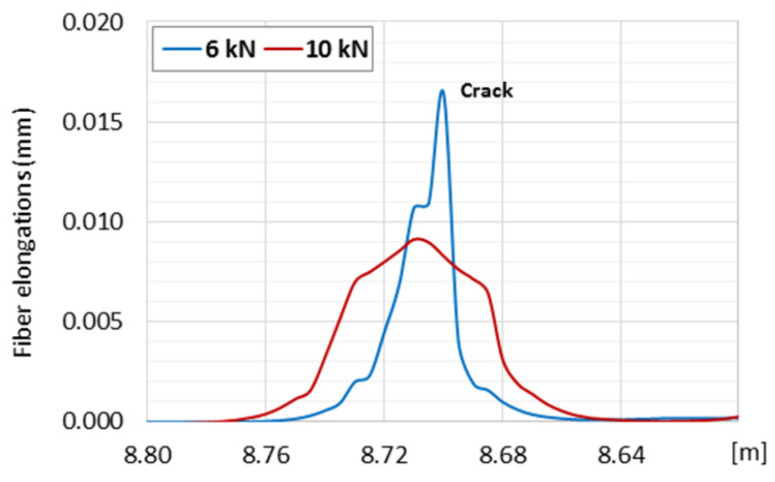
The effect of slippage inside layered sensing cable when measuring crack opening in concrete member under axial tension. As the load increases, the crack peak is reduced, instead of higher. Blue—force of 6 kN, Red—force of 10 kN. When measured correctly, an increase in force should result in increase in the measured strain peak.

Layered design of sensing cables allows these layers to slip uncontrolled in relation to each other; thus, it is not possible to unambiguously transfer strain to the internal measuring fiber in the desired strain range. An intuitive example showing the influence of layer slippage effect on the final strain results is presented in Figure 4 [6]. In the present example the telecom fiber SM9/125 in tight jacket was used (it was embedded into concrete beam). The layered structure of the cross section caused local slippages which disturb strain transfer in the vicinity of the cracks. The local strain peak corresponding to crack was decreased while increasing the force. Such an effect is not acceptable for reliable and accurate data analysis. Slippage was observed even when analyzing small cracks less than 0.05 mm (Figure 4) [6]. The wider the cracks, the more probable the slippage effect is. Such slippage can also occur between the sensing cable with smooth external surface and the surrounded material, such as concrete.

Plastic (nonlinear) effects including slippage between intermediate layers but also yielding of steel components in the cable (responsible for mechanical memory of events) make it impossible to monitor the current deformation state of the structure, e.g., closing of cracks under the impact of external factors. Such characteristics of layered cables prevent their application for monitoring the strengthened collector described hereafter in the article.

## 5. Analysis of Available Solutions: Monolithic Strain Sensors

**Figure 5:**
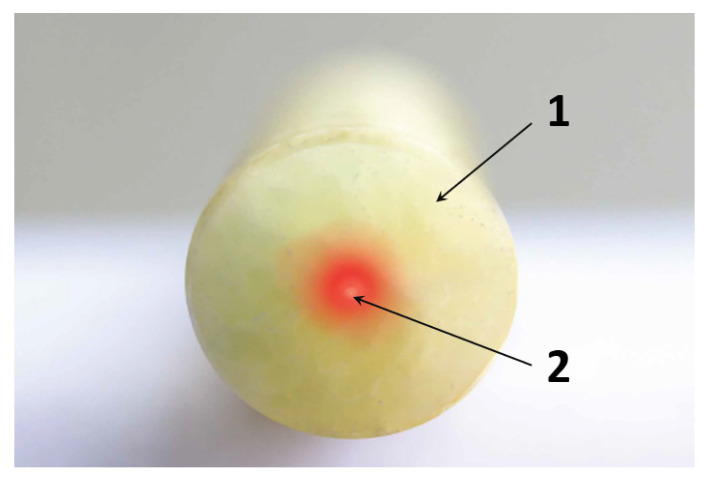
Cross-section of DFOS measurement sensor used for strain measurement. 1—monolithic core, 2—optic fiber [7].

The other possible solution is distributed fiber optic sensors (DFOS) designed specially to measure strains of the monitored structure. The sensors consist of a monolithic, composite core in which a measuring optical fiber in its primary coating is integrated during production process (pultrusion). There are no intermediate layers or external coatings, what allows for unambiguous, predictable transmission of strain from the surrounding material (e.g., concrete) to the measuring fiber inside the core. Figure 5 illustrates a monolithic cross-section of a typical DFOS strain sensor.

**Figure 6:**
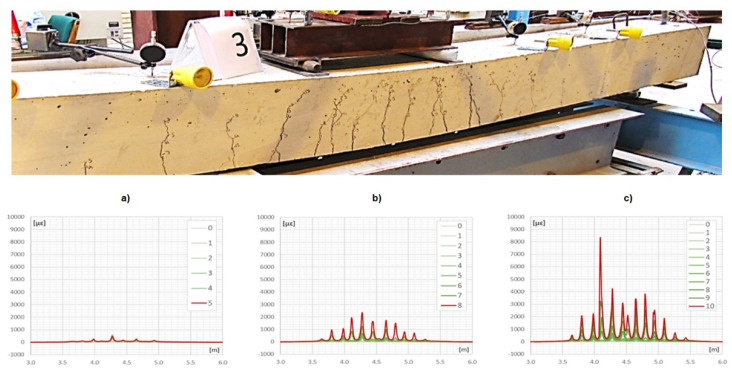
Crack analysis in reinforced concrete beam using monolithic DFOS sensors. Sensor was embedded inside the beam at the height of main bottom reinforcement (courtesy by [8]). Graphs show strains development during increasing the load (local peaks correspond to cracks near to the bottom surface); (**a**) initial phase; (**b**) middle phase; (**c**) final phase.

Figure 6 shows the example results of crack analysis based on indications of DFOS monolithic sensors during 4-point bending test of a reinforced concrete beam. Local strain extremes (peaks) registered in length domain correspond to all the cracks, which were also clearly observed by unarmed eye. Three plots show the strain distributions for three selected load steps during research: in the initial, middle and final phase (before beam failure). The increase in load caused the increase in the measured strain peaks without any smoothing caused by the slippage effect, which has been observed previously inside the layered sensing cable.

Additional advantages of the DFOS fiber optic sensors include a wide measurement range of up to ±4%, ribbed or braided external surface that ensures good integration of the sensor and the surrounding concrete as well as a high resistance to the installation process and environmental conditions.

Given the requirements: unambiguous strain measurement, no slippage between layers, possibly wide measurement range and resistance to sewage collector operating conditions, monolithic composite DFOS sensors have been selected for the system.

## 6. DFOS-Based Structural Health Monitoring System

**Figure 7:**
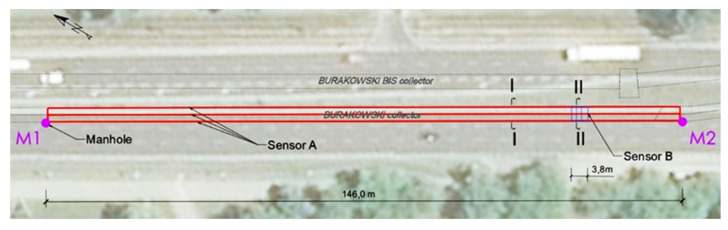
Sensor locations along the collector.

The monitoring covered the renovated segment of the Burakowski sewage collector, specifically the section where the structural damage was most significant. The length of the pilot section equipped with sensors was about 146 m (Figure 7). The sensors were brought to the ground surface through two inspection chambers M1 and M2 (Figure 7) located in the road lane at opposite ends of the installation to allow placement of the measuring equipment outside the collector.

**Figure 8:**
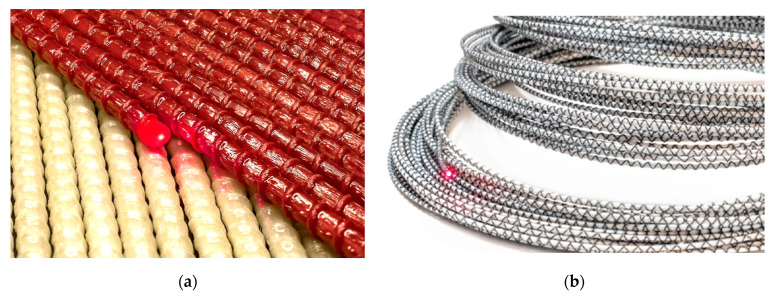
Sensors used in the monitoring system. (**a**) Sensor A—EpsilonRebar, (**b**) Sensor B—EpsilonSensor [8].

Two types of sensors were installed in the monitored section—see Figure 8. The first one (type A—EpsilonRebar) is constructed from glass-fiber core with a measuring range of ±2%. The second one (Sensor B—EpsilonSensor) is based on a polyester fiber core with a measuring range of ±4%. The optical fiber is fully integrated with the sensors’ core during their production process (pultrusion).

The sensor locations were determined by reference to similar sewage collector and tunnel monitoring systems, including those performed in 2016 as a part of the Grand Paris Express [9] project and currently being implemented for the entire investment, which is expected to be completed in 2030 [10]. Four loops in the most weakened part of the collector section were implemented in a manner similar to the design of the system used in the Royal Mail Underground Tunnel in London [11]. Experiences of other researchers have proven the robustness of fiber-optic sensors for structural monitoring under difficult conditions in cross-sections of tunnels [12].

**Figure 9:**
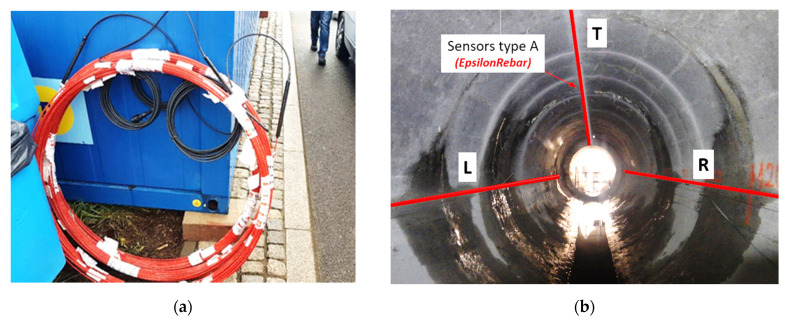
(**a**) EpsilonRebars delivered to the site in coils; (**b**) arrangement of DFOS sensors (type A) in the cross section of the collector.

**Figure 10:**
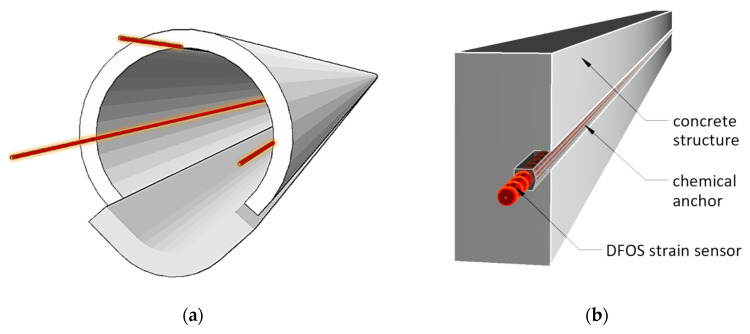
Spatial visualizations: (**a**) longitudinal sensors arrangement; (**b**) scheme of installation inside the groove filled with chemical anchor.

**Figure 11:**
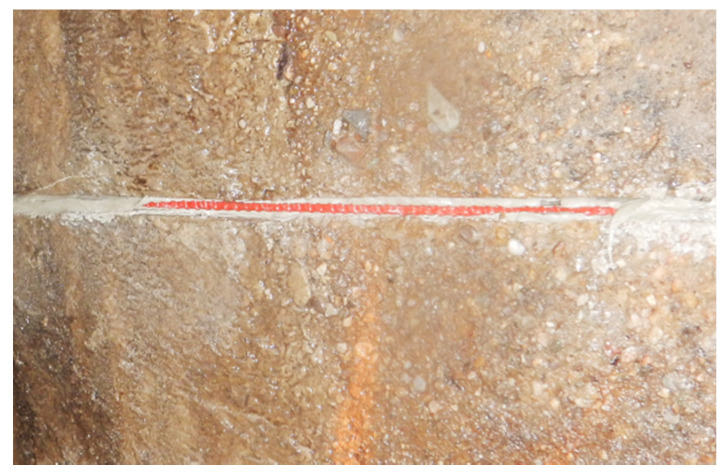
View of the sensor on the collector wall inside the groove during filling it with chemical anchor.

All sensors with appropriate lengths were delivered to the site in coils (Figure 9a). Three longitudinal measurement lines were installed along the axis of the collector: L, T and R (at 9, 12 and 3 o’clock positions respectively–Figure 9b and Figure 10a). Sensors were placed in prepared longitudinal grooves with dimensions of approximately 8 × 12 mm (width × depth), made in the wall of the existing collector. The grooves were finally filled with chemical anchor—see Figure 10b and Figure 11.

Sensors installed in grooves are protected from mechanical damage during installation of the GRP modules and properly integrated with the existing concrete casing.

**Figure 12:**
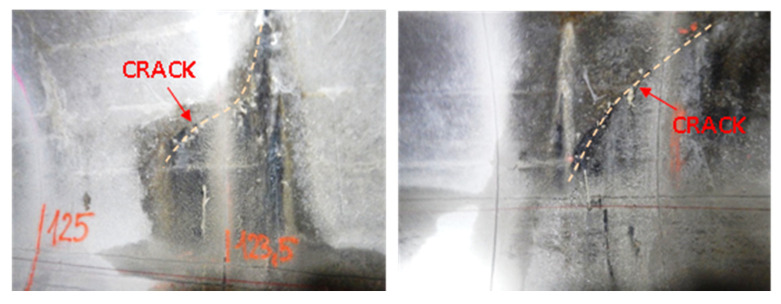
Examples of diagonal cracks of a collector concrete structure.

**Figure 13:**
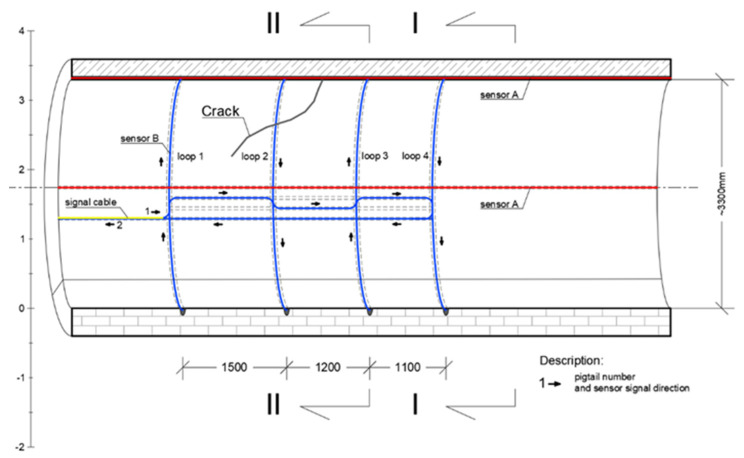
Scheme of DFOS sensors (type B) installation in the cracked section of collector.

**Figure 14:**
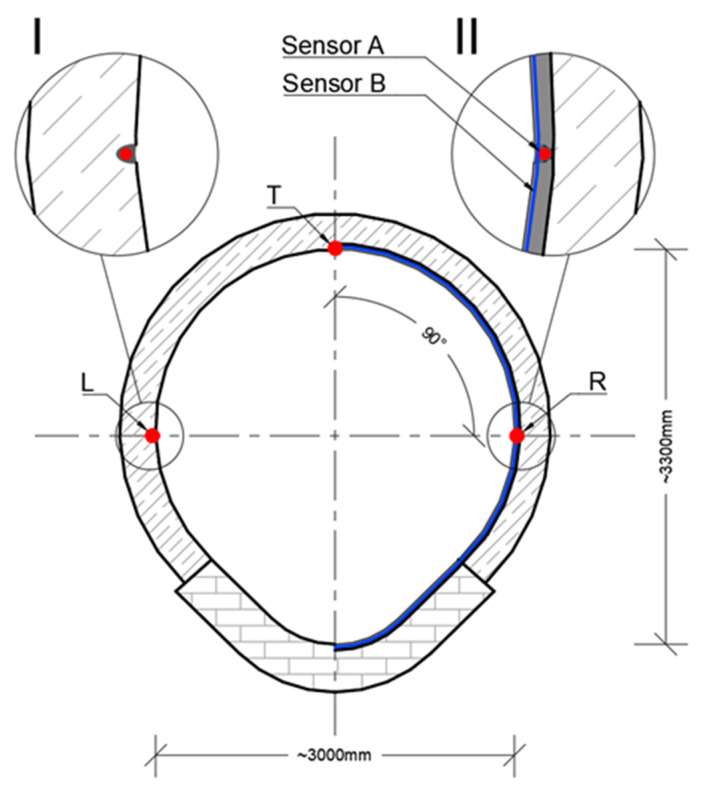
Scheme of DFOS sensors arrangement in walls of the existing collector. I—section outside the loops, II—intersection of sensors A and B.

The other type of sensor—EpsilonSensor (B)—was used to measure circumferential strains between 120–125 m of the monitored section, where diagonal cracks in the collector walls were visible with an unarmed eye (Figure 12). EpsilonSensor with elastic modulus equal to E = 3 GPa is a flexible tool and could be freely and precisely shaped in the grooves located along the circumference in selected safety-critical cross sections. The measuring range of EpsilonSensor is equal to ±4% and allows to monitor even large cracks without risk of sensor breakage. A 62.6 m long sensor was installed on loops in four measuring sections (Figure 12 and Figure 13). The sensor was installed in grooves similar to EpsilonRebars described before. Scheme of DFOS sensors arrangement in walls of the various section existing collector is presented in Figure 14: I—section outside the loops, II—intersection of sensors A and B.

## 7. Example DFOS Measurement Results

The measurement sessions were performed using an optical backscatter reflectometer (OBR) from Luna Innovations. This device is based on Rayleigh scattering [5]. The readings were taken in both standard and extended mode with spatial resolution 10 mm and 100 mm respectively. Data presented hereafter correspond to 10 mm spatial resolution.

Strain measurements were taken during the strengthening works, including the stage before GRP modules were placed inside the collector (zero reading), after the GRP modules were placed (before grout injection) and finally after grout injection. DFOS monitoring allowed to observe structural response of the collector under gradual load changes.

**Figure 15:**
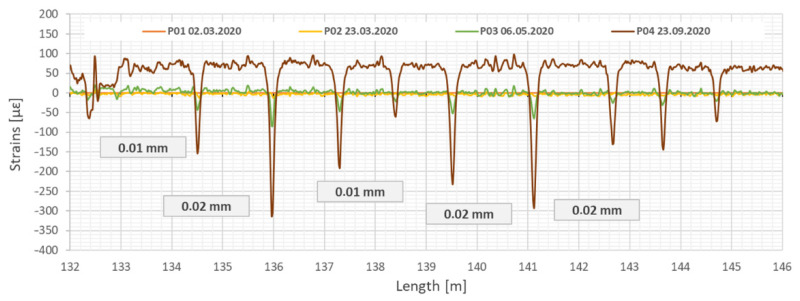
Strain profiles measured by sensor (T) along the test section showing the primary discontinuities in the concrete collector closing during strengthening process. Example section from 132 to 146 m. Measurement P01—after installation of GRP panels, P02—during grout injection, P03—after completed grout injection, P04—after filling the collector with sewage.

A particularly significant effect observed due to the DFOS measurements was revealing the original structure of the concrete collector casing, related to technological breaks during its construction. Increasing load on the concrete casing during the renovation works revealed cracks caused by technological breaks and changes in their widths were carefully analyzed during this process (Figure 15). Concrete casing sections originally constructed were from 2.2 to 2.9 m long. The application of monolithic fiber optic strain sensors allowed to identify all discontinuities in the collector structure and to observe their development over time (most of these cracks were undetectable by unarmed eye).

Figure 15 shows example results of measurements taken with longitudinal sensor located at the top (T) of the collector. Under the additional load related to strengthening process (including dead weight of GRP panels, grout injection and sewage during operation), the cracks were closing (decreasing their width). The observed changes were small (less 0.02 mm), but they still were clearly detectable.

Based on the strain measurements acquired from three lines of EpsilonRebars, vertical displacements of the collector caused by the strengthening works (including dead weight of the composite panels and mortar injection) were determined. The analysis was based on the trapezoidal method [13,14,15,16]. The main operation rule of this method is to divide the structure into the discrete sections (trapezoids) with the base equal to the spatial resolution of distributed measurements. The height of the trapezoid is equal to the distance between the upper and lower DFOS sensors. In the present case, strain values were obtained from the top (T) and arithmetic mean from left (L) and right (R) sensors (see also Figure 11). Deformations of individual trapezoids are calculated using only geometrical relationships and they are summed up over the length to obtain displacement profiles. This method does not require the knowledge on material properties of the monitored structure. Detailed drawings, equations and further explanations of the method applied are presented in paper [13].

**Figure 16:**
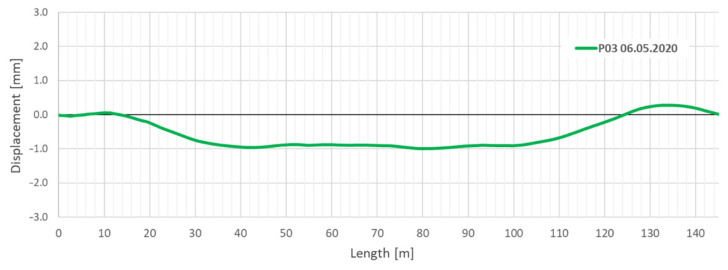
Relative vertical displacements (change in shape) of the collector after installation of GRP panels and grout injection.

The mid-length displacement (settlement) of the collector determined after the grout injection process relative to its ends was about 1 mm, as shown in Figure 16.

## 8. Conclusions

The application of fiber optic measurements using DFOS sensors with a monolithic core allowed to create an effective monitoring system for a concrete collector reinforced with GRP panels, operating in difficult and variable ground and water conditions. A wide spectrum of information acquired from the sensors may, in the future, contribute to the reduction of required functional inspections carried out directly by the operator. In particular, the system allowed to:

1. Identify discontinuities of the collector’s concrete casing (cracks) and observe their development over time. In the analyzed case study, deformations were caused by the dead weight of GRP segments, grout injection used to fill the gap between the new composite panels and old collector, as well as the thermal-shrinkage effect of the cement grout;
2. Determine relative displacements (changes in shape) of the collector along the whole measured section, both in the vertical and horizontal plane. The relative displacements determined in the vertical plane after strengthening were about 1 mm;
3. Measure the temperature distribution along the entire length of the collector. Such measurements can be used to detect leaks through the collector casing. They are also used for thermal compensation, which is one of the key aspects during long term structural health monitoring. In the presented case study, thermal compensation was performed due to two approaches:
  - application of additional optical device based on Raman scattering to obtain temperature profiles along the entire collector. EpsilonRebars were used simultaneously as the temperature sensors;
  - application of additional spot measurements at the start and end point of the collector to obtain reference temperature readings.

In addition, the analysis of changes in the collector strains along its length recorded using distributed fiber optic sensors (DFOS) can provide important information on the structural performance of the collector. An indication of change in tendency (even if changes are minor) at the initial stage of operation helps to plan and carry out remedial work as soon as possible. The ultimate goal is to create a situation where a properly designed, constructed and operated structural health monitoring system will enable an objective assessment of the technical condition of a facility over its entire service life, and inform the users of any abnormalities. The application of distributed fiber optic sensors is a promising solution for these purposes. DFOS can be successfully used to detect and observe phenomena that are typically local in nature, such as steel yielding, junction cracking, concrete cracking, leakages etc. None of the commercially available, conventional spot techniques provide similar capabilities.

## Data Availability

The data presented in this study are available on request from the corresponding author.
